# Altered Functional Connectivity of Cognitive-Related Cerebellar Subregions in Well-Recovered Stroke Patients

**DOI:** 10.1155/2013/452439

**Published:** 2013-06-19

**Authors:** Wei Li, Tong Han, Wen Qin, Jing Zhang, Huaigui Liu, Ying Li, Liangliang Meng, Xunming Ji, Chunshui Yu

**Affiliations:** ^1^Department of Radiology and Tianjin Key Laboratory of Functional Imaging, Tianjin Medical University General Hospital, Tianjin 300052, China; ^2^Cerebrovascular Diseases Research Institute, Xuanwu Hospital of Capital Medical University, Beijing 100053, China

## Abstract

The cerebellum contains several cognitive-related subregions that are involved in different functional networks. The cerebellar crus II is correlated with the frontoparietal network (FPN), whereas the cerebellar IX is associated with the default-mode network (DMN). These two networks are anticorrelated and cooperatively implicated in cognitive control, which may facilitate the motor recovery in stroke patients. In the present study, we aimed to investigate the resting-state functional connectivity (rsFC) changes in 25 subcortical ischemic stroke patients with well-recovered global motor function. Consistent with previous studies, the crus II was correlated with the FPN, including the dorsolateral prefrontal cortex (DLPFC) and posterior parietal cortex, and the cerebellar IX was correlated with the DMN, including the posterior cingulate cortex/precuneus (PCC/Pcu), medial prefrontal cortex (MPFC), DLPFC, lateral parietal cortices, and anterior temporal cortices. No significantly increased rsFCs of these cerebellar subregions were found in stroke patients, suggesting that the rsFCs of the cognitive-related cerebellar subregions are not the critical factors contributing to the recovery of motor function in stroke patients. The finding of the disconnection in the cerebellar-related cognitive control networks may possibly explain the deficits in cognitive control function even in stroke patients with well-recovered global motor function.

## 1. Introduction 

Ischemic stroke is one of the leading causes of motor disability in adults, while most patients experience a certain degree of recovery of motor function. The mechanisms of motor recovery after stroke have been extensively investigated especially using the neuroimaging techniques [1–6]. However, the contribution of cerebellum to motor recovery after stroke is a subject of much debate. The contralesional cerebellum is a part of the affected motor network and has reciprocal connections with the ipsilesional sensorimotor cortex. In subcortical stroke, contralesional cerebellar hypometabolism [7–9] and atrophy [10] are reported and have been ascribed to the damage of anatomical connections by lesions [11, 12]. Initially the affected cerebellum has been shown to exhibit greater activation during performing a motor [13] or a tactile exploration task [14] with the affected hand. Subsequently, this activation decreased gradually and this was correlated with functional recovery [15]. In a longitudinal analysis of the executive motor network, the betweenness centrality (a measure evaluating the importance of a node in brain network) of the affected cerebellum was negatively correlated with the recovery of motor function [16].

Beyond the motor function, the cerebellum is also critical for cognitive control [17–23]. Sang et al. [24] have showed detailed resting-state functional connectivity (rsFC) patterns of cerebellar subregions in healthy subjects. In that study [24], the cerebellar crus II was typically connected with the frontoparietal network (FPN) which is associated with cognitive control and the cerebellar IX was mainly connected with the default-mode network (DMN), which is deactivated during cognitively demanding tasks. Other studies have also identified that cerebellum IX contributes to the DMN [24, 25], cerebellar VII (including crus II) contributes to higher-level processes [21, 24, 26], and they were activated during cognitive paradigm [27, 28]. 

Although the recovery of motor function in stroke patients is associated with the reconstruction of the motor network, it is suggested that the recovery of motor function is a process of relearning the motor skills [29–31]. The cerebellar subregions (crus II and lobule IX), as important nodes in relearning the motor skills, may contribute to motor recovery after stroke. If the hypothesis is correct, we predict that the rsFCs between the cerebellar subregions (crus II and lobule IX) and the cerebral regions of the FPN and DMN will increase in stroke patients with good recovery in global motor function; in contrast, the decreased rsFCs may indicate functional impairment of the two cerebellum-related cognitive control networks (FPN and DMN) in these stroke patients. In the present study, we aimed to investigate the rsFC changes of these two cognitive-related cerebellar subregions in 25 well-recovered subcortical ischemic stroke patients to clarify their roles in motor recovery after stroke. 

## 2. Method and Materials

### 2.1. Subjects

**Figure 1:**
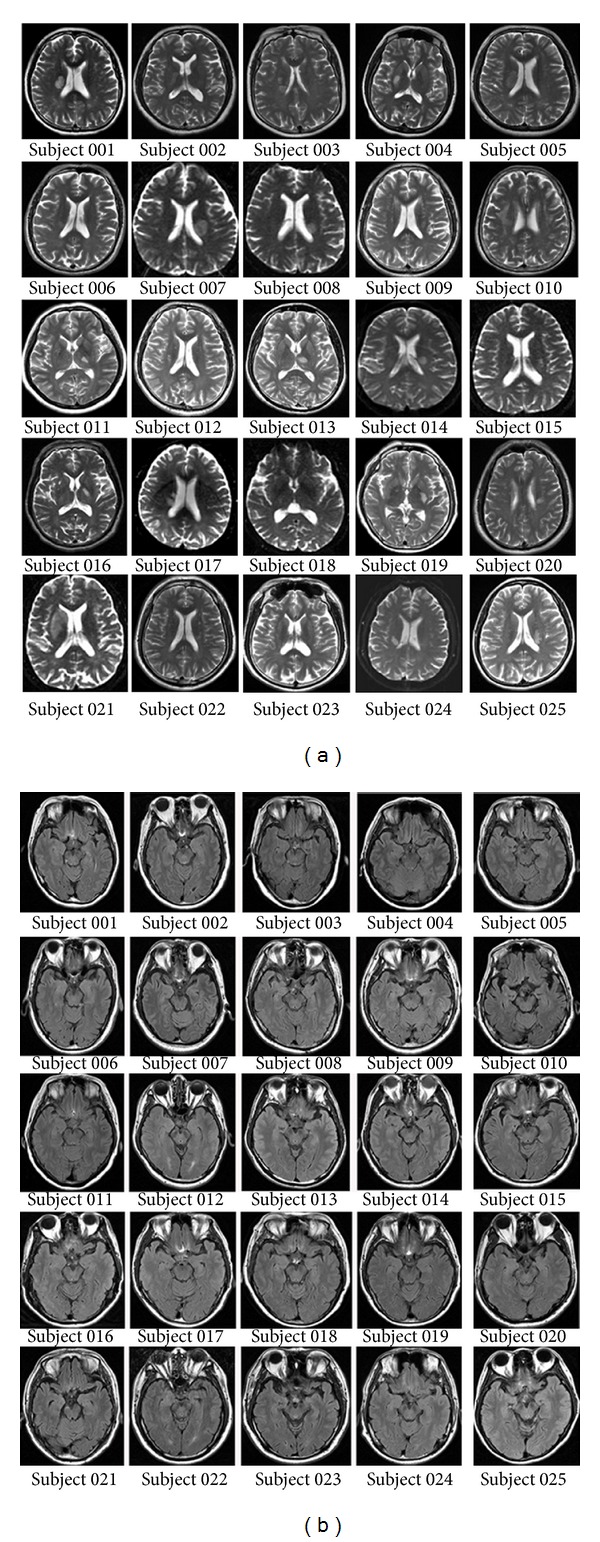
(a) Lesion locations of stroke patients on axial T2-weighted images acquired at the stroke onset when stroke patients were first enrolled in our hospital. (b) The morphology and signals of the brainstem of every stroke patient.

The experiment was approved by the Ethical Committee of Tianjin Medical University General Hospital and written informed consent was obtained from each subject before the study. Inclusion criteria were as follows: (1) first-onset stroke; (2) manifested motor deficit at stroke onset; (3) unilateral single lesion involving the internal capsule and neighboring regions; (4) being right-handed; (5) 6 months or more since the time of infarct; (6) with good recovery of motor function, the upper extremity Fugl-Meyer test (UE_FMT) of >60, and the whole extremity Fugl-Meyer test (WE_FMT) of >90; (7) compliance to carry out the experiment. Exclusion criteria were as follows: (1) recurrent stroke after first onset; (2) with any other brain abnormalities other than the infarct lesion, either history of neurological or psychiatric disorders, or did any of them experience subsequent symptomatic stroke; (3) severe white matter hyperintensity revealed by T2W images; (4) history of drug dependency or psychiatric disorders; (5) conscious disturbance or uncooperative; (6) head motion parameters of fMRI data satisfied a maximum displacement of <2 mm or a maximum rotation of <2.0°. According to the criteria, 25 patients (7 females; mean age: 56.2 years; range: 42–72 years) were finally included in this study (Figure 1, Table 1). Twenty-two age-matched healthy controls (11 females; mean age: 57.2; range: 47–74 years) were also recruited as controls who reported no history of psychiatric or neurological problems and the head motion parameters of fMRI data satisfied a maximum displacement of <2 mm or a maximum rotation of <2.0°.

### 2.2. Data Acquisition

Sagittal 3D T1-weighted images were acquired by a brain volume (BRAVO) sequence with the following parameters: repetition time (TR)/echo time (TE) = 8.1/3.1 ms; field of view (FOV) = 256 × 256 mm^2^; matrix = 256 × 256; slice thickness = 1.0 mm, no gap; 176 slices. The resting-state fMRI data of all the subjects were obtained using a gradient-echo single-shot echo-planar imaging sequence (GRE-SS-EPI) with the following parameters: TR/TE = 2000/30 ms; slice thickness = 3 mm, 1 mm gap; matrix = 64 × 64; FOV = 240 × 240 mm^2^; 38 transverse slices; 180 volumes. During fMRI scans, all subjects were instructed to keep their eyes closed, to stay as motionless as possible, to think of nothing in particular, and not to fall asleep.

### 2.3. Data Preprocessing

Before the data preprocessing, we have flipped the imaging data from left to right about the midsagittal line for patients with lesions on the left hemisphere. For all patients, the right side was the ipsilesional (affected) hemisphere while the left side was the contralesional (unaffected) hemisphere. The resting-state fMRI data were preprocessed using Statistical Parametric Mapping (SPM8, http://www.fil.ion.ucl.ac.uk/spm/). The first 10 volumes from each subject were discarded to allow the signal to reach equilibrium and the participants to adapt to the scanning noise. The remaining 170 volumes were corrected for acquisition time delay between slices. Then, head motion parameters were estimated; none of the 47 subjects had a maximum displacement of >2 mm or a maximum rotation of >2.0°. In this step, the framewise displacement (FD) was also calculated to characterize the instantaneous head motion of each volume. The remaining data set was then normalized into the Montreal Neurological Institute (MNI) EPI template and resampled into 3 × 3 × 3 mm^3^ voxels. Thereafter, some nuisance variables were regressed from the BOLD data, including the averaged signals of the ventricular, white matter, the whole brain, six head realignment parameters obtained by rigid body head motion correction, and the temporal derivatives of each of these realignment parameters. Because recent studies found that signal spike caused by motion also significantly contaminates the final resting-state fMRI results even after regressing out the realignment parameters, in this study, we also removed the spike volumes by generating an individual vector when the FD of specific volume exceeded 0.5. Next, a band-pass frequency filter (0.01–0.08 Hz) was applied to reduce low-frequency drift and high-frequency noise. Finally, the filtered BOLD images were spatially smoothed with an isotropic Gaussian kernel of FWHM = 8 mm.

### 2.4. Definition of Cerebellar ROIs

**Figure 2:**
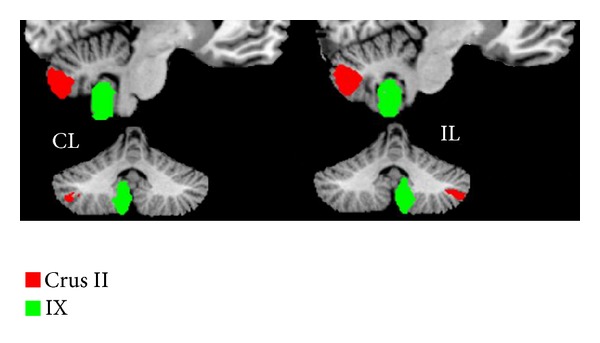
The bilateral seed regions of the cerebellar subregions of crura II and IX extracted from the probabilistic cerebellar atlas with a threshold of 50% minimum probability.

The bilateral cerebellum subregions of crura II and IX were extracted from the Probabilistic cerebellar atlas [32] with a threshold of 50% minimum probability (Figure 2).

### 2.5. The rsFC Analysis of Cerebellar Crura II and IX

For each individual dataset, Pearson's correlation coefficients between the mean time series of each seed region and that of each voxel of the whole brain were computed and converted to *z* values using Fisher's *r*-to-*z* transformation to improve the normality. Then, individuals' *z*-values were entered into a random-effect one-sample *t*-test in a voxel-wise manner to identify brain regions that showed significant positive correlations with the seed region. A corrected threshold of *P* < 0.05 was derived from a combined threshold of *P* < 0.01 for each voxel and a cluster size >65 voxels which was determined by the AlphaSim program in the AFNI software (parameters: single voxel *P* = 0.01, 5000 simulations, FWHM = 8 mm, cluster connection radius = 5 mm, with gray matter mask, http://afni.nimh.nih.gov/).

### 2.6. Intergroup Comparisons of the rsFCs of the Cerebellar Crura II and IX

A two-sample *t*-test was performed to quantitatively compare differences in rsFCs of the two cerebellar subregions between controls and patients with age and gender being treated as covariates of no interest. A corrected threshold of *P* < 0.05 was derived from a combined threshold of *P* < 0.01 for each voxel and a cluster size >31 voxels which was determined by the AlphaSim program in the AFNI software (parameters: single voxel *P* = 0.01, 5000 simulations, FWHM = 8 mm, cluster connection radius = 5 mm, with a mask of gray matter regions showing positive rsFC with each cerebellar ROI, http://afni.nimh.nih.gov/).

### 2.7. The Analysis of Subgroup Stroke Patients Group

To reduce the influence of the inconsistency of lesion location on our results, we selected 11 stroke patients with lesions involving the posterior limb of internal capsule, through which the corticospinal tracts pass. Using the same method, we run the one-sample *t*-test in stroke subgroup patients and the two-sample *t*-test between stroke subgroup patients and healthy controls.

## 3. Results

### 3.1. Demographic and Clinical Information

Twenty-five subcortical stroke patients with well-recovered motor function and twenty-two normal controls were recruited in the present study. The clinical and demographic data of the stroke patients are listed in Table 1. The lesion location of each patient was demonstrated on axial T2W images, which were acquired at the stroke onset when stroke patients were first enrolled in our hospital (Figure 1(a)). Compared with the healthy controls, stroke patients did not show any significant differences in both age (two-sample *t*-test: *t*_45_ = −0.50, *P* = 0.62) and gender (chi-square test: *χ*_1_^2  ^ = 2.40, *P* = 0.12). As shown in Table 1, the duration from stroke onset to MRI scanning ranged from 11 months to 64 months (mean value: 29.0 ± 16.4 months). The ischemic lesions involved the internal capsule regions and surrounding structures, including the internal capsule, lentiform nucleus, caudate nucleus, corona radiate, and thalamus; thirteen out of the 25 stroke patients had infarct lesion in the right hemisphere and 12 in the left hemisphere. Except for 3 patients that failed to measure the MMSE score, the remaining 22 patients showed relative normal cognitive status (MMSE ranged from 26 to 30, mean 28.3 ± 1.3). The Fugl-Meyer test showed that both the motor functions of upper and lower extremities were well recovered (minimum 62 for upper extremities and 94 for all extremities).

### 3.2. Whole-Brain rsFC Patterns of the Cerebellar Crus II

**Figure 3:**
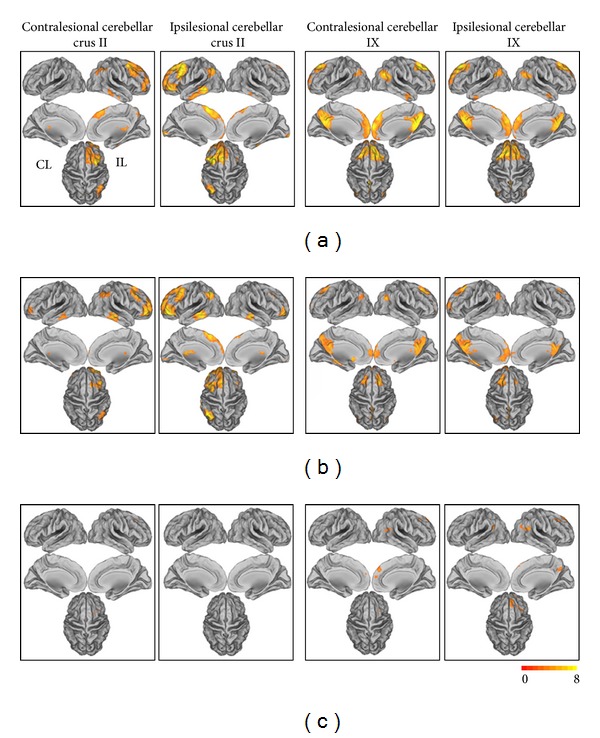
The rsFC patterns of the cerebellar subregions in healthy controls and stroke patients (total: *n* = 25) and the rsFC differences between stroke patients and healthy controls (*P* < 0.05 corrected). (a) The rsFC patterns of the cerebellar subregions in healthy controls; (b) the rsFC patterns of the cerebellar subregions in stroke patients; (c) the differences in the rsFCs between stroke patients and healthy controls.

We only focused on the positive rsFCs of each cerebellar subregion because the functional significance of the negative rsFC is a matter of debate that whether the negative connectivity is an artifact of the global signal regression [33, 34] or reflects dynamic, anticorrelated functional networks [35]. In healthy controls, the cerebellar crus II was mainly correlated with the contralateral FPN, including the dorsolateral prefrontal cortex (DLPFC) and posterior parietal cortex (see Figure 3(a) and Table 2). Stroke patients showed similar rsFC patterns of the cerebellar crus II but the strengths and spatial extent were slightly different from those of healthy controls (see Figure 3(b) and Table 2). 

### 3.3. Whole-Brain rsFC Patterns of the Cerebellar IX

In the healthy controls, the cerebellum IX showed strong rsFCs with the DMN mainly including the posterior cingulate cortex/precuneus (PCC/Pcu), medial prefrontal cortex (MPFC), DLPFC, lateral parietal cortices, and anterior temporal cortices (see Figure 3(a) and Table 2). Stroke patients showed similar rsFC patterns of the cerebellar IX but the strengths and spatial extent were lower relative to those of healthy controls (see Figure 3(b) and Table 2).

### 3.4. Altered rsFC Patterns of Cerebellar Crus II in Stroke Patients

Compared with healthy controls, stroke patients showed decreased rsFC between the contralesional cerebellar crus II and the ipsilesional DLPFC and between the ipsilesional cerebellar crus II and the contralesional caudate nucleus (Figure 3(c) and Table 2). In contrast, there was no significant increase in rsFC between the cerebellar crus II and any of cortical or subcortical forebrain regions.

### 3.5. Altered rsFC Patterns of Cerebellar IX in Stroke Patients

Compared with healthy controls, stroke patients showed decreased rsFC between the contralesional cerebellar IX and the ipsilesional MPFC, DLPFC, and lateral parietal cortices. Stroke patients also had decreased rsFC between the ipsilesional cerebellar IX and the ipsilesional PCC/Pcu and prefrontal areas and the bilateral lateral parietal cortices (Figure 3(c) and Table 2). In contrast, there was no significant increase in rsFC between the cerebellar IX and any of cortical or subcortical forebrain regions.

### 3.6. The Analysis of Subgroup of Stroke Patients

**Figure 4:**
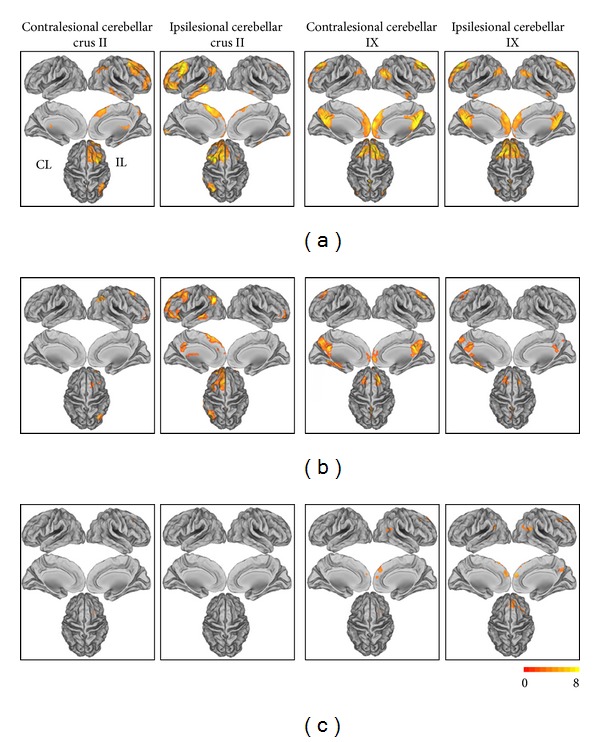
The rsFC patterns of the cerebellar subregions in healthy controls and stroke patients (subgroup: *n* = 11) and the rsFC differences between stroke patients and healthy controls (*P* < 0.05 corrected). (a) The rsFC patterns of the cerebellar subregions in healthy controls; (b) the rsFC patterns of the cerebellar subregions in stroke patients; (c) the differences in the rsFCs between stroke patients and healthy controls.

The results of the subgroup of stroke patients are shown in Figure 4. Both the rsFCs of stroke patients and intergroup differences were similar to those obtained from all stroke patients.

## 4. Discussion

### 4.1. The rsFC Patterns of the Cerebellar Crura II and IX

In the present study, we found that the cerebellar crus II was mainly correlated with the brain regions of the contralateral FPN, including the DLPFC and posterior parietal cortex, which is well consistent with previous findings on the FPN [24, 25, 36–38]. A transneuronal tracing study also supports these findings by showing that crus II has reciprocal connections with the DLPFC [39]. We also found that the cerebellar lobule IX was mainly correlated with brain regions of the DMN, including the PCC/Pcu, MPFC, lateral parietal cortices, DLPFC, and anterior temporal areas. This finding was in agreement with previous rsFC studies [24, 25, 37, 38]. 

### 4.2. The Relationship between the FPN and DMN

The FPN and DMN are the two most important cognitive-related functional networks in the human brain. The FPN is involved in cognitive control, such as working memory [40, 41] and executive control function [27], whereas the DMN is implicated in self-referential processing [42], conscious awareness [43], mind wandering [44], and retrieval and manipulation of episodic memories and semantic knowledge [45]. During the performance of cognitively demanding tasks, the FPN typically shows increases in activation, whereas the DMN shows decreases in activation [46, 47]. During resting state, the FPN and DMN are anticorrelated with each other and the strength of this anticorrelation was associated with cognitive performance [48, 49]. Consequently, we selected the cerebellar crura II and IX as subregions of interest to represent the cerebellar-related cognitive control regions.

### 4.3. No Increased rsFC of the Cerebellar Crura II and IX Subregions in Stroke Patients

The motor recovery of stroke patients is commonly thought of as a relearning process of the motor skill [29–31]. Various rehabilitation approaches have been used to improve skill reacquisition of the impaired arm [50] and enhanced clinical outcomes depend on both the training intensity and task specificity [51]. The cognitive condition or ability for the recovery of motor function has been extensively reported [52, 53] and cognitive strategy-based interventions have been shown to have beneficial effects on the recovery of motor function in stroke patients [50, 54]. Thus the motor skill reacquisition may need the cognitive control system either during the spontaneous recovery or the rehabilitation training. This inference is also supported by the finding that motor improvements of stroke patients were related to better memory, mental flexibility, and planning abilities [50]. 

In the present study, we aimed to test whether the rsFCs of the cognitive-related cerebellar subregions have beneficial effects on the recovery of motor function in stroke patients. Unexpectedly, we did not find any significantly increased rsFC of either the cerebellar crus II or the cerebellar lobule IX in stroke patients. These negative results suggest that the rsFCs of the cognitive-related cerebellar subregions do not contribute to the recovery of motor function in stroke patients. However, this negative finding does not exclude the possibility of the involvement of the cognitive-related brain regions in the motor recovery process because several studies have shown that cognitive-related brain regions such as the hippocampus were enlarged in chronic stroke patients [55] or after rehabilitation training [56].

### 4.4. Decreased rsFC of the Cerebellar Crura II and IX Subregions in Stroke Patients

In stroke patients, the cerebellar crus II showed decreased rsFCs with brain regions of the FPN and the cerebellar lobule IX exhibited decreased rsFC with brain regions of the DMN. These findings suggest that the decreased rsFCs of the cerebellar crus II and the cerebellar lobule IX are the hallmark in these stroke patients with well-recovered global motor function. It has been reported that subcortical stroke not only impaired the motor function but also impaired the cognitive function [57, 58] through damaging the white matter fibers that are important for cognitive processing. Volumetric studies have revealed the extensive atrophy in the bilateral frontal, parietal, and insular areas, especially the affected hemisphere [1, 58] which are important for cognitive function. The functional disconnection within the DMN has been reported in stroke patients and the disconnection was associated with the poststroke depression severity [59]. Additionally, the impaired FPN was also reported in stroke patients [60]. Beyond the previous findings of the involvement of the DMN and FPN, we provide evidence that the rsFCs between the cognitive-related cerebellar subregions (crus II of the FPN and lobule IX of the DMN) and cerebral regions of their corresponding networks (FPN and DMN) are also impaired in stroke patients even though they recovered well in global motor function. In addition, we found that most of cerebral regions showing impaired rsFCs with the two cerebellar subregions were located in the affected cerebral cortex, which further supports our inference that these decreased rsFCs may represent the impaired cognitive function in these stroke patients. However, the lack of specialized cognitive assessments impedes us to determine the relationship between these rsFC impairments and the behavioral performance in these stroke patients. Further studies should be performed to determine the relationship. 

## 5. Limitation

A major weakness of this study is the inclusion of various groups of stroke, such as different lesion location and different clinical deficits. Although we reperformed the analyses in a subgroup of stroke patients with the involvement of the corticospinal tract and found similar results, further studies with larger sample size and more homogeneous clinical characteristics are needed to verify our findings. Another limitation of this study is the lack of evaluation of sensory and cognitive deficits, which should be done in future studies.

## 6. Conclusion 

In the present study, we analyzed the alterations of the rsFCs of the cerebellar crura II and IX in stroke patients with well-recovered global motor function. We did not find any significantly increased rsFCs of these cerebellar subregions in stroke patients, suggesting that the rsFCs between cognitive-related cerebellar subregions (crus II of the FPN and lobule IX of the DMN) and cerebral regions of their corresponding networks (FPN and DMN) do not experience plastic changes that may facilitate the motor recovery in subcortical stroke patients with good recovery of their global motor function. In contrast, stroke patients showed decreased rsFCs between these cerebellar subregions and their corresponding functional networks. These findings may represent functional disconnection within both the FPN and DMN even in well-recovered stroke patients, which may be associated the cognitive deficits in stroke patients.

## Figures and Tables

**Table 1:** Clinical information of stroke patients.

ID	Sex	Age (year)	Duration (month)	Lesion location	MMSE	FMT	
						UE	WE
BG001	F	65	63	Right PLIC, CR	29	62	94
BG002	M	62	41	Right PLIC, GIC	27	66	100
BG003	F	63	48	Right CR, PLIC, LN	30	65	98
BG004	F	52	64	Right CR, PLIC, LN	30	65	99
BG005	M	53	37	Right CR, PLIC, LN	/	62	95
BG006	M	65	37	Right CR	28	66	98
BG007	M	59	41	Left CR, PLIC, LN	29	64	98
BG008	M	49	40	Left CR, PLIC, LN	28	65	98
BG009	M	60	30	Left CR	26	66	100
BG010	F	72	41	Right CR, PLIC, LN	26	65	98
BG011	F	55	24	Left Th	26	66	100
BG012	M	49	24	Right CR, LN	30	66	100
BG013	M	42	24	Left Th	28	66	100
BG014	M	50	18	Left CR, PLIC	27	66	100
BG015	M	52	22	Left CR, PLIC	29	66	100
BG016	M	58	52	Left PLIC	29	66	100
BG017	M	65	20	Right CR, PLIC, LN	28	65	99
BG018	F	63	14	Right Th	28	66	100
BG019	M	55	11	Left PLIC, LN	30	66	100
BG020	M	47	13	Left CR	/	66	100
BG021	M	58	14	Right CR, Cau, ALIC	/	66	100
BG022	M	63	13	Left CR, PLIC	27	66	100
BG023	M	45	11	Right CR	30	66	100
BG024	M	49	13	Right CR, PLIC	30	64	96
BG025	F	53	11	Left CR, PLIC, LN	28	66	99

ALIC: anterior limb of internal capsule; Cau: caudate; CR: corona radiate; GIC: genu of internal capsule; LN: lenticular nucleus; PLIC: posterior limb of internal capsule; Th: thalamus.

**Table 2:** The rsFCs of the cerebellar crura II and IX in both of the control and patient groups and significant group differences in these rsFCs between healthy controls and stroke patients.

Seed regions	Connected regions	Group difference	Peak *t* values	Peak coordinate MNI (*x*, *y*, *z*)	Cluster size (voxels)
CL_crus II	IL_DLPFC	∗	4.23	30, 18, 39	74
	IL_LPC				
	IL_Pcu				
	IL_ITG				
	IL_MTG				
	CL_ITG				
	CL_DLPFC				

IL_crus II	B_DLPFC				
	B_MPFC				
	IL_ITG				
	CL_LPC				
	CL_MTG/ITG				
	CL_PCC/Pcu				
	CL_Cal				
	CL_Cau	∗	4.11	−12, 6, 15	40
	CL_Th				

CL_IX	B_PCC/Pcu				
	B_MCC				
	IL_MPFC	∗	3.42	6, 48, 33	80
	IL_ACC				
	IL_MTG/MOG				
	IL_LPC	∗	3.18	54, − 57, 18	44
	IL_DLPFC	∗	3.98	33, 24, 42	38
	CL_DLPFC				
	CL_LPC				
	CL_MPFC				

IL_IX	IL_MPFC	∗	4.35	18, 57, 21	241
	IL_LPC	∗	4.14	54, − 54, 21	103
	IL_DLPFC	∗	3.97	36, 24, 45	77
	IL_MTG				
	IL_PCC/Pcu	∗	3.30	15, − 54, 33	51
	CL_ParaHP				
	CL_LPC	∗	3.95	−51, − 57, 24	66
	CL_PCC/Pcu				

ACC: anterior cingulated cortex; Cal: calcarine; Cau: caudate; CL: contralesional hemisphere; DLPFC: dorsolateral prefrontal cortex; IL: ipsilesional hemisphere; ITG: inferior temporal gyrus; LPC: lateral parietal cortex; MCC: middle cingulate cortex; MOG: middle occipital gyrus; MPFC: the medial part of prefrontal cortex; MTG: middle temporal gyrus; ParaHP: parahippocampal; PCC: posterior cingulate cortex; Pcu: precuneus; Th: thalamus. The star (∗) represents that the rsFC shows significant group difference between stroke patients and healthy controls.
